# Collagen from Cartilaginous Fish By-Products for a Potential Application in Bioactive Film Composite

**DOI:** 10.3390/md16060211

**Published:** 2018-06-15

**Authors:** Emna Ben Slimane, Saloua Sadok

**Affiliations:** 1 Laboratory of Blue Biotechnology & Aquatic Bioproducts, Institut National des Sciences et Technologies de la Mer (INSTM), INSTM, 28, rue 2 mars 1934, Salammbô, Tunis 2025, Tunisia; benslimaneemna@gmail.com; 2 Institut National Agronomique de Tunisie (INAT), Université de Carthage, Tunis 1082, Tunisia

**Keywords:** cartilaginous fish by-products, collagen, chitosan, composite films, properties

## Abstract

The acid solubilised collagen (ASC) and pepsin solubilised collagen (PSC) were extracted from the by-products (skin) of a cartilaginous fish (*Mustelus mustelus*). The ASC and PSC yields were 23.07% and 35.27% dry weight, respectively and were identified as collagen Type I with the presence of α, β and γ chains. As revealed by the Fourier Transform Infrared (FTIR) spectra analysis, pepsin did not alter the PSC triple helix structure. Based on the various type of collagen yield, only PSC was used in combination with chitosan to produce a composite film. Such film had lower tensile strength but higher elongation at break when compared to chitosan film; and lower water solubility and lightness when compared to collagen film. Equally, FTIR spectra analysis of film composite showed the occurrence of collagen-chitosan interaction resulting in a modification of the secondary structure of collagen. Collagen-chitosan-based biofilm showed a potential UV barrier properties and antioxidant activity, which might be used as green bioactive films to preserve nutraceutical products.

## 1. Introduction

During the last decades, the increased consumer awareness of the nutritional value of fish and seafood and the shift towards more processed fishery products in convenient form; has generated larger quantities of by-products accounting for up to 70% of the volume of fish and shellfish [1]. In most cases, such biomasses, which include skin, head, bones and viscera, cause serious economic and ecological issues. However, such biomass is currently of high interest to researchers and industry as it represents a valuable source of compounds with high added value such as proteins, lipids, enzymes, and polysaccharides. 

Animal body contains high amount of collagen constituting around 30% of the total amount of protein in vertebrates [2]. Actually, 27 types of collagens have been identified and collagen type I is the most frequent one and is known as fibrillar collagen and plays a structural role by contributing to the molecular architecture, shape and mechanical properties of skin tissues [2,3]. Due to its excellent properties (non-toxicity, low antigenicity and allergenicity, biocompatibility, the ability of film-forming and biodegradability), collagens are utilised in various fields such as medical, pharmaceutical and cosmetics industries, and also as materials for food packaging [4,5,6]. 

At the start of its use, collagen was mainly extracted from porcine and bovine sources. Later, people started to show reticence toward this practice due to religious background, beside the proliferation of bovine spongiform encephalopathy (BSE) [7] which represented a source of hazard contamination for the extracted protein [8,9]. As a consequence, several researchers have been interested in marine collagen as an alternative because of the absence of disease transmission and dietary restriction [10].

Collagen has been studied and characterised from various marine sources, mainly from marine invertebrates such as cuttlefish [11], octopus [12], squid [13], jellyfish [14], starfish [15], sea urchin [16], sea cucumber [17] and also of sponges which represent the key of their complex structure and integrity [18]. In marine vertebrate organisms, such interest was rather oriented to fish by-products including scales [19], skin [20,21], swim bladder [22], bone [23] and cartilage [24]. 

For their extractions, collagens are commonly solubilized in organic acid, generally acetic acid which causes the protonation of collagen polypeptides and consequently the repulsion between the tropo-collagen leading to enhanced collagen solubility [25]. However, such procedure referred to Acid Solubilised Collagen (ASC) gives generally low collagen yield. Therefore, research was oriented to enzymatic extraction to increase collagen solubilisation with pepsin being among the most efficient enzyme. Thus, pepsin provokes not only the cleavage of the collagens teleopeptide region maximising their solubility, but also the hydrolysis of non-collagenous proteins increasing collagen purity. In this case, pepsin maximise the extraction yield of collagens while reducing their antigenicity [26,27]. 

The common coastal smooth-hound *Mustelus mustelus,* is an abundant species of the genus *Mustelus* in the Mediterranean Sea where it is regularly caught all over the year either as by-catch or as targeted species [28,29]. The consumption of such species generates significant amounts of waste that may be used as source to extract substance of interest such as collagen. 

To our knowledge, the extraction of collagen from the skin of *Mustelus mustelus* and its valorisation has never been reported. Therefore, the aim of this work was to extract and characterize collagen using two methods. In a first step, the isolation of collagen was elaborated using acetic acid, which allows a better solubilisation of the molecule followed in a second step, by an enzymatic extraction using pepsin.

Our second objective was to elaborate a biodegradable film using collagen, however following the extraction process (alkali then acid process), the collagen molecule loses its strong mechanical strength compared to the native form [30]. To overcome such issue, we blended collagen with another natural polymer such as chitosan derived from chitin known as the second most abundant polysaccharide after cellulose. Thus, chitosan has attracted much attention for its biodegradability, biocompatibility, bacteriostatic and fungistatic activities as well as for its texturizing properties and its ability to film forming [31]. Therefore, we used chitosan as an adjuvant to elaborate composite film.

## 2. Results and Discussion

### 2.1. Collagen Characterization

#### 2.1.1. Collagens Electrophoretic Patterns

**Figure 1:**
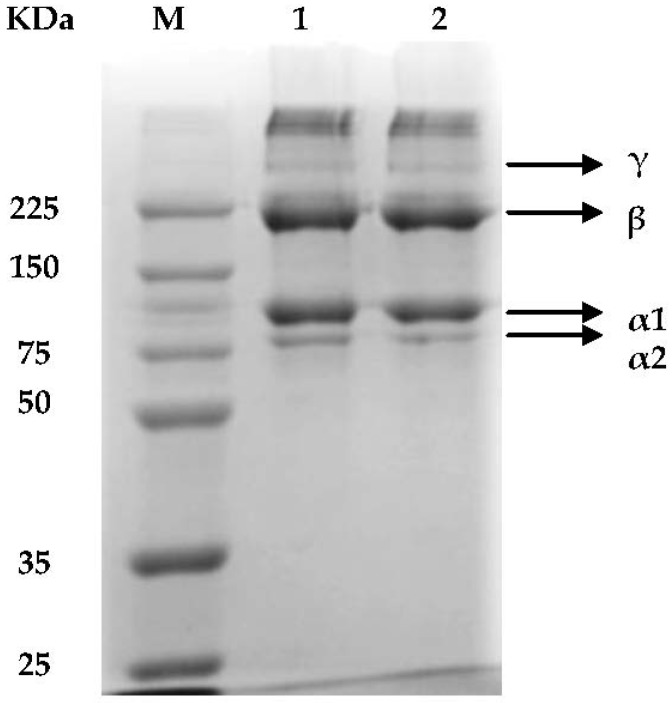
Sodium dodecyl sulfate polyacrylamide gel electrophoresis (SDS-PAGE) of (1): acid soluble collagen (ASC) and (2): pepsin soluble collagen (PSC) from hound-smooth skin M: high molecular weight marker (KDa).

The electrophoretic patterns of collagens from smooth-hound skin (ShS) performed under denaturing condition are presented in Figure 1 and showed that there are no differences between the ASC and pepsin solubilised collagen (PSC). 

As native collagen molecule is constituted of three polypeptide chains (α-chains) organised in a triple-helix, the denaturising sodium dodecyl sulfate (SDS) break the H-bonds yielding peptides [32]. In both types of ShS-collagen, the α (α1, α2) and their cross-linked dimer β-chains are the major components with low content of γ-chain. Thus, the electrophoresis mobility and subunit composition may suggest that ASC and PSC isolated from ShS should most likely be classified as type I collagen. The SDS-PAGE, revealed two bands of chains α1 and α2 with a molecular weight of about 101 kDa and 83 kDa respectively; however with different intensities (α1 intensity higher than α2 by approximately ratio 2:1). Such results suggest that α1 is formed by 2 subunits as collagen type I characterised by the existence of 2 identical subunits of α1 and one of α2 [33].

Additionally, high molecular weight (MW) components, β-chains were clearly detected in both ShS-ASC and PSC with a mean molecular weight of 226 kDa. Such results are in conformity with several findings reported for other elasmobranches skins of brownbanded bamboo shark [34], skate [21] and shark [35].

#### 2.1.2. Peptide Mapping

**Figure 2:**
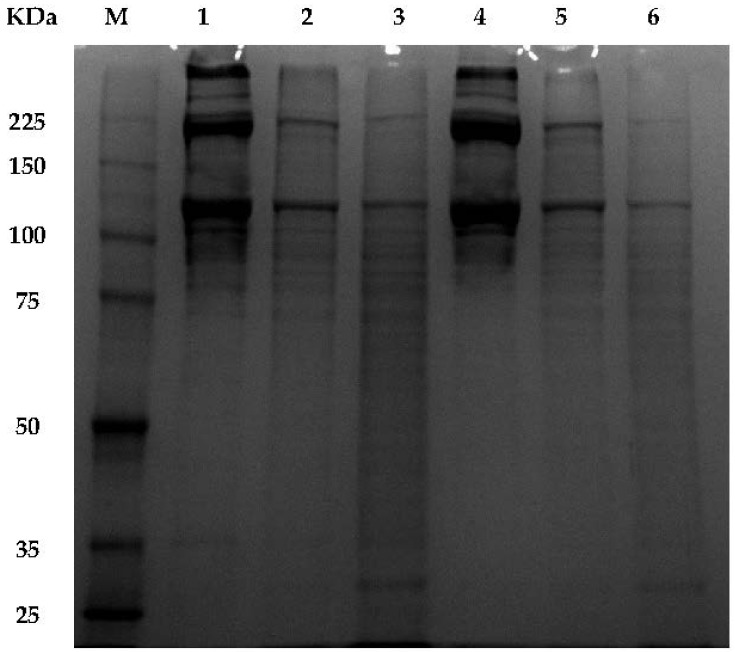
Peptide mapping of acid soluble collagen ASC and pepsin soluble collagen PSC digested by *Lysyl endopeptidase* with different hydrolysis time 1: ASC, 2: ASC-5 min, 3: ASC-25 min, 4: PSC, 5: PSC-5 min, 6: PSC-25 min from hound smooth skin, M: high molecular weight marker (KDa).

The ShS-ASC and PSC were markedly digested by Lysyl endopeptidase which cleaves peptide bonds at the carboxyl side of lysyl residues [36]. Generally, band intensity of major components α, β and γ of ShS-ASC and PSC decreased after digestion and degraded into smaller peptides with molecular weight ranging from 100 to 13 kDa (Figure 2). 

When comparing the effect of hydrolysis duration, an enhanced enzymatic hydrolysis was found with an incubation time of 25 min for ShS-ASC and PSC. This was evidenced by the appearance of higher number of peptides bands with low molecular weight (Figure 2, lines 3 and 6); and a decreased band’s intensity for PSC collagen. This might be caused by the pepsin action on the telopeptide region inducing its cleavage and thus facilitating the changes in configuration, which may favour the hydrolysis by lysyl endopeptidase.

#### 2.1.3. Viscosity Measurement

**Figure 3:**
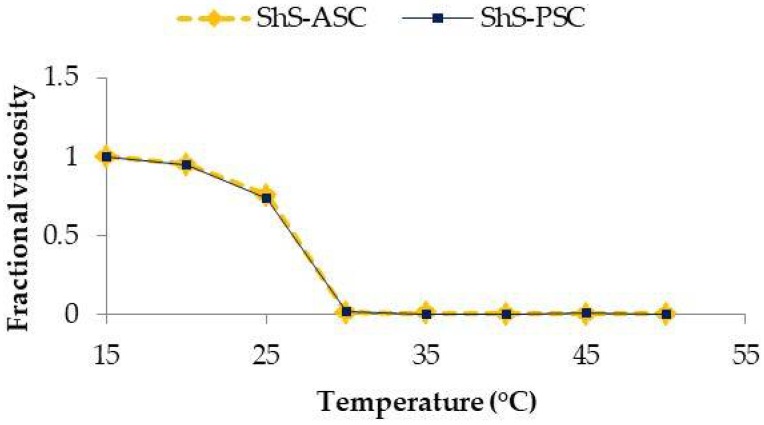
Change in fractional viscosity with temperature of acid soluble collagen (ShS-ASC) and pepsin soluble collagen (ShS-PSC) from the skin of hound smooth.

The temperature of denaturation (Td) of ShS-ASC and PSC, referred as the temperature at which the variation in viscosity is half completed, was calculated from a plot of temperature-induced variation in viscosity (Figure 3).

The viscosities of both collagens were higher at temperature ranging from 15–20 °C then decreased with increased heating up to 25 °C. 

The Td’s of ASC and PSC were 26.68 °C and 26.66 °C respectively. The similarity in the denaturation temperature may be related to the resemblance of the major peaks wavelength of ASC and PSC [37]. Such values were comparable to Td reported for collagen from other marine species such as the Japanese sea bass (26.5 °C) [23], edible jellyfish exumbrella (26.0 °C) [14], chub mackerel (25.6 °C), bullhead shark (25.0 °C) [23] but lower than porcine skin collagen (37.0 °C) [11]. However, the Td’s of both ASC and ASC smooth hound skin were higher than those of Spanish marckel skin (15.12 and 14.66 °C, respectively) [38].

#### 2.1.4. Ultraviolet Spectrophotometric Analysis

**Figure 4:**
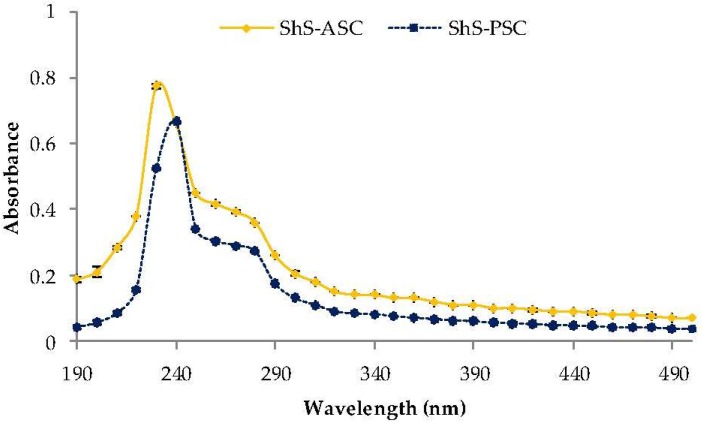
Ultraviloet-Visible spectra of acid soluble (ShS-ASC) and pepsin soluble collagen (ShS-PSC) from the skin of hound smooth.

The ShS-ASC and PSC collagens exhibited maxima absorbencies at 235 nm and 240 nm respectively (Figure 4), which is in agreement with the maximum absorption of the collagen molecule (230 nm) [39]; principally due to the *n* → π* transitions of the peptide band C=O [40].

Unlike other protein types no peak was detected at 280 nm, suggesting that ASC and PSC collagens have low amount of aromatic residues such as tyrosine and phenylalanine [41]. These results are in line with those previously found in fish skin collagens [34,42,43,44] and confirm the effectiveness of the alkaline treatment for the removal of non-collagenous proteins.

#### 2.1.5. Fourier Transform Infrared Spectra of Collagens

In order to determine the isolated collagens type; Fourier transform infrared technique was used to detect the vibrational modes of bands and individual chemical groups in the extracted collagens [45]. 

**Figure 5:**
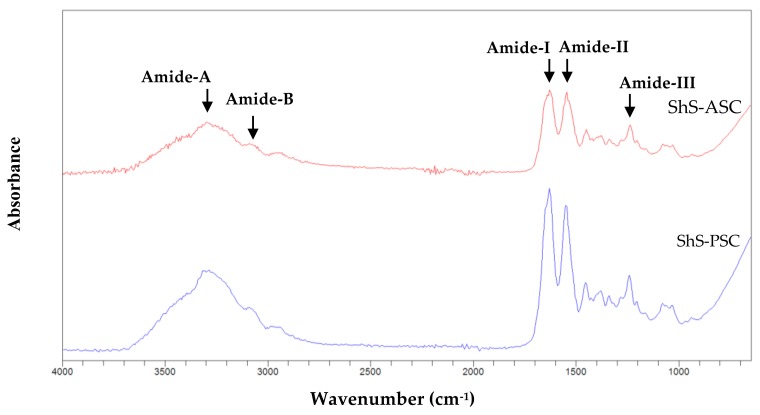
Fourier Transform Infrared spectra of ASC (acid soluble collagen) and PSC (pepsin soluble collagen) from the skin of hound smooth fish.

The ShS-collagen’s Fourier Transform Infrared (FTIR) spectra represented in Figure 5, showed that the amide A band of ASC and PSC originated from the stretching vibrations of N–H group were found at 3293.57 and 3296.16 cm^−1^ respectively, although it commonly appears in the range of 3400–3440 cm^−1^ [19,46]. These shifts to lower frequencies means that the collagen NH groups of the samples were involved in hydrogen bonding, which help to stabilize the collagen triple helix structure. The absorption peak of amide B, related to asymmetrical stretch of CH_2_ [47], appeared at 2942 cm^−1^ for ASC and at 3092.05 cm^−1^ for PSC. Such results are concordant with that reported for the collagen extracted from the skin of splendid squid [13].

The amide I band mainly associated with stretching vibrations of carbonyl groups (C=O bond) along the polypeptide backbone [48], was depicted at 1629.6 cm^−1^ and 1629.5 cm^−1^ for ASC and PSC respectively, this amide is actually a sensitive marker for peptide secondary structure [49]. 

The PSC and ASC amide II bands were situated at wavenumbers of 1548.95 and 1545.17 cm^−1^ respectively; while the ASC and PSC-amide III bands were located at wavenumbers of 1237.94 and 1239.84 cm^−1^, respectively. The amide II band represent N=H bending vibrations coupled with C=N stretching vibration [50], and amide III peak reflects intermolecular interactions in collagen, including peaks from C–N stretching and N–H deformation from amide linkages. It is also related to absorptions resulting from wagging vibrations from CH_2_ groups from the glycine backbone and proline side-chains [51].

The Infra-Red absorption (IR) ratios between amide III and 1454 cm^−1^ band for the ASC and PSC fractions were found around 1 (0.95 and 1.08 respectively); indicating the persistence of the triple helix structure within the extracted collagen [52].

Such detailed description allowed to conclude that the slight differences observed between the ASC and PSC structure may be caused by pepsin treatment which has the effect to remove the telopeptide region, whereas the similarity of IR ratios may suggest that pepsin had no influence on the structure of the collagen triple helix. 

### 2.2. Biofilms Mechanical and Functional Properties

#### 2.2.1. Mechanical Properties

**Figure 6:**
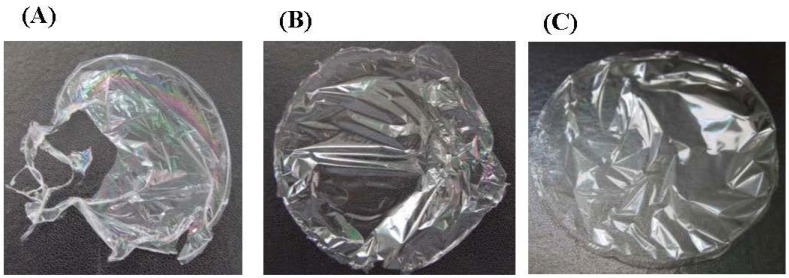
Pictures of (**A**) pure collagen film CO, (**B**) bi-composite Collagen-chitosan film and (**C**) pure chitosan film CH.

One of the laboratory objectives was to elaborate green edible biofilm using collagen from seafood by-products without any chemical addition and at low collagen percentage taking in consideration the cost of its production. However, when using skin *M. mustelus* collagen solution at 0.1%; the film was too fragile to allow any mechanical properties analysis (Figure 6A). Therefore, blending collagen with another polymer such as chitosan known for its high film-forming ability and lower cost was necessary to enhance the biofilm compactness (Figure 6B). Beside the resulting film showed akin aspect to the pure chitosan film (Figure 6C). The results suggest that the aggregation occurring between the collagen molecules of the film matrix was filled by the dispersed chitosan enhancing the cohesion between the various complexes within the adsorbed layer as shown in other study [53].

Such assumption is reflected by the thickness of the elaborated biofilms which showed different values as summarized in Table 1. The highest thickness value was noted in the pure chitosan film (17.15 μm) and decreased with an increased collagen ratio into the chitosan solution. Similar result were reported for the composite films using chitosan and collagen from the unicorn leatherjacket skin [54]. In the present study, higher chitosan ratio (75%) had no significant effect (*p* > 0.05) on film’s thickness (Table 1) which showed similar smoothness and compactness. Such results suggest that the chitosan charge density was sufficiently high at the 50% ratio to assure the complexation between protein and polysaccharide knowing that the degree of compactness of the gel network is regulated by the polysaccharide charge density [55].

The tensile strength (TS) was also affected by degree of protein/polysaccharide ratio. Thus, the pure chitosane film value was 70.52 MPa and decreased with the addition of collagen. The increased chitosan proportion in composite films increased significantly (*p* < 0.05) the tensile strength from 55.42 MPa for C_75_ to 66.28 MPa for C_50_. This is not only due to the interactions between chitosan and collagen molecules by electrostatic force but also by hydrogen bonding [56,57]. Similarly, the percentage of elongation at break (EAB) increased significantly (*p* < 0.05) with the incorporation of collagen in composite films from 4.25% in pure chitosane films to 4.49–5.67% in C_75_ and C_50_ respectively. This phenomenon is attributed to the hydrophilic properties of collagen which provides a certain increase in the hydration degree of the film giving an upper elongation at break value [58].

The comparison of the mechanical properties of the obtained composite films with those found in literature gives contradictory interpretations (Table 2) due to several factors including species origin (habitat, diet), the collagen amino acid composition [59], the protocol of extraction and the polymers ratio (collagen/chitosan). 

Similarly, the mechanical properties of chitosan-based films are affected by various parameters such as the chitosan deacetylation degree, their molecular weight, as well as the conditions of film preparation (pH of the film-forming solution, the water content, and the drying conditions) [60,61,62,63].

In this study, composite films (C_50_, C_75_) exhibited tensile strength of similar or higher values than commercial films (LDPE 13%, HDPE 26%, Hydroxypropyl cellulose 15%) and collagen-chitosan-based films reported in other studies (Table 2). However, the elongations at break values of the composite films (C_50_, C_75_) were much lower than those of commercial films, since there is an inverse relationship between TS and EAB [64].

#### 2.2.2. Water Solubility

The highest water solubility was observed in pure collagen films (32.14%) and decreased significantly (*p* < 0.05) with the incorporation of increased chitosan percentage (24.55% and 17.64% for the C_50_ and C_75_ films respectively; Table 3).

Actually, the film’s resistance to water is owed to the hydrophobic nature of chitosan molecule and to the covalent bond “amide bond” which has the effect of reducing the polarity of the films [54].

Indeed, an edible film must have both good resistance to water in order to preserve the integrity of the product [69] and a good ability to dissolve when ingested by the consumer and degrade naturally if it released into the environment [70].

However, the increase of water resistance of composite films could not be perceived as an advantage since high solubility cannot shield the product from humidity and water loss [71].

#### 2.2.3. Optical Properties—Colour, Opacity and Light Transmittance of the Films

The film colour is a key element in the consumer’s appreciation of the product since this parameter has a direct influence on the product appearance, especially when the film is to be used for packaging. For the various elaborated films, the highest (*p* < 0.05) L*-value (Lightness) and a*-value (redness/greenness) and the lowest b*-value (yellowness) and ΔE* (colour difference) were recorded in pure collagen films (Table 2). Thus, incorporation of chitosan induced a significant decrease in the lightness (*p* < 0.05), particularly in the films with the highest concentration of chitosan C_75_, making them more yellowish. This may be due to the reaction of Maillard which took place between the carbonyls groups of chitosan and collagen amino groups [72].

The highest b*-value was observed in pure chitosan film (CH) and as described by Kurek, et al. [73], this parameter (b*-value) defines the natural colour of chitosan, Yellow, which is related to the presence of β-1-4 linked 2-amino-2-deoxy-d-glucopyranose repeating units [74].

In addition to the colour of the film, transparency is also a very influential parameter in relation to the acceptability of the product. Generally, a clear film is more attractive clearly displaying the contents of the product. For all films, the light transmission in UV-Visible range was negligible at 200 nm, regardless of types and concentrations of chitosan. Collagen film exhibit the highest transmission at 280 nm but after the addition of chitosan, transmission decreased from 72.6 % for collagen pure film to 63.3% and 50.6% for composite films C_50_ and C_75_ respectively (*p* < 0.05).

**Figure 7:**
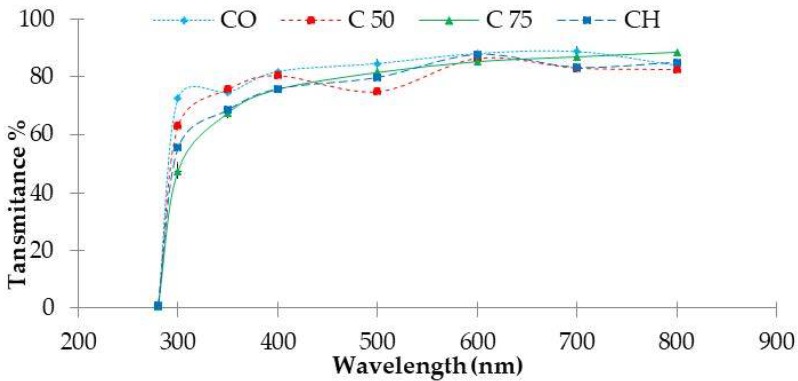
Optical transmission spectra of collagen films. CO: pure collagen film; CH: pure chitosan film; C_50_: Collagen-chitosan film 50%:50%, C_75_: Collagen-chitosan film 25%:75%.

The results show that when increasing the concentration of chitosan, composite films have better UV barrier properties. For instance, it makes these collagen-chitosan films usable as preventive materials against loss of nutrients and discoloration caused by the lipid oxidation [75]. The transmission of visible light at 400–800 nm, was superior to 80% in pure collagen film (CO), and was significantly higher (*p* < 0.05) than that of the collagen-chitosan composite films (Figure 7).

#### 2.2.4. Fourier Transform Infrared Spectra of Composite Films

For the composite biofilms, FTIR was used to detect the new interactions between collagen and chitosan and to identify the nature of the new linkages between both molecules.

**Figure 8:**
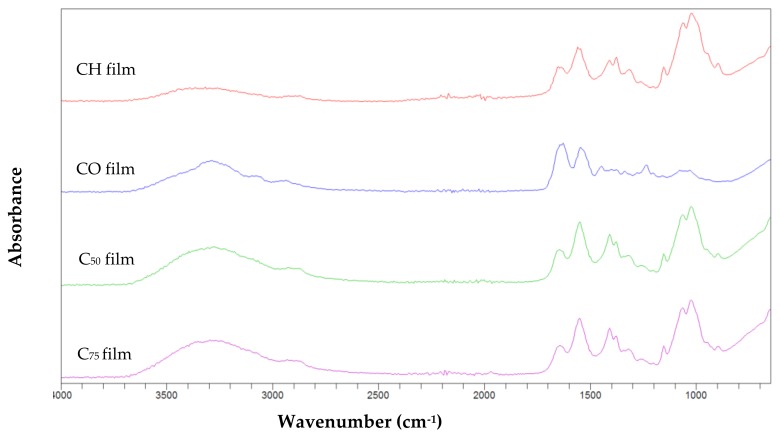
Fourier transform infrared spectra of different collagen films, CO: pure collagen film; CH: pure chitosan film; C_50_: Collagen-chitosan film 50%:50%, C_75_: Collagen-chitosan film 25%:75%.

The FTIR spectra revealed the characteristics of the specific bands corresponding to functional groups in all films (Figure 8). 

The CO, C_50_, and C_75_ composite films; displayed amide I bands at the wavenumbers of 1641.2, 1645.5 and 1642 cm^−1^ respectively. The shift to higher wavenumber was a result of a structural rearrangements occurring in the film structure with a strong affinity between the collagen and chitosan. 

The amide I peak in CH film at the wavenumber of 1642.6 cm^−1^ was assigned to C=O stretching of N-acetyl group [76]. The amide II band was found at approximately the same wavenumber in CO film (1543.9 cm^−1^) and C_50_ film (1543.6 cm^−1^) but at lower peak compared to C_75_ (1550.61 cm^−1^) and CH films (1550.8 cm^−1^).

At amide III region, the addition of the chitosan had as result a significant decrease in the wavenumber from 1239.9 for CO film to 1016.9 and 1022.7 cm^−1^ for C_50_ and C_75_ films respectively. This shift induced by chitosan addition suggested some interaction between the CH_2_ side chains of collagen molecule with that of chitosan molecules [67].

As shown in Figure 8, amide A peak of collagen film decreased after incorporation of chitosan from 3294.5 to 3250.4 cm^−1^ for C_50_ film and to 3288.5 cm^−1^ for C_75_ film, this suggests the loss of hydrogen bonding between water and collagen by chitosan interaction [77]. 

Similarly, a shift to lower frequency was noticed for amide B from 2941.9 for collagen film to 2932.2 and 2925.42 cm^−1^ for collagen-chitosan films C_50_ and C_75_. As previously reported, when the CH group of a peptide was involved in a hydrogen bond with other polymer, the position of amide B moved to lower frequency.

The FTIR spectra clearly indicated that interactions between the two polymers have taken place and the secondary structure of collagen had been changed by chitosan incorporation.

#### 2.2.5. Radical Scavenging Activity of Films

The radical scavenging activities, DPPH (1,1-diphenyl-2-picrylhydrazyl) of the different type of films are showed in Table 4.

Pure collagen film exhibited the highest radical scavenger with 30.8%. However, the addition of chitosan into the collagen solution, induced a significant decrease (*p* < 0.05) of the DPPH radical-scavenging ability of the composite films to values of 23.91% and 19.77% for C50, C75, respectively, when compared to pure collagen film. This result might be explained by the reaction that took place between residual free amino groups of chitosan and free radicals which may form stable macromolecular radicals and ammonium groups [81].

The scarcity of data on the DPPH- scavenging activity of collagen-based films, did not allow a comparative study. However, when comparing with other work such as cuttlefish gelatin-based films, CO film exhibited similar DPPH- scavenging values (Table 4). Regarding the composite films (C50, C75), scavenging activity was less or similar to other composite films (Table 4).

## 3. Experimental Section

### 3.1. Raw Materials

Smooth-hound (*Mustelus mustelus*) were purchased from a local market in La Goulette, Tunisia and brought immediately to the laboratory. Fish were thoroughly washed with cold tap water and manually de-skinned as occurs in the marked. The cleaned fish skins were cut into pieces (approximately 1 cm × 1 cm) and subjected to a pre-treatment for collagen extraction.

### 3.2. Pretreatment of Fish Skin

To remove pigments and non-collagenous proteins; the fish skins pieces (FSPs) were immersed into a solution of NaOH (0.1 M, ratio skin: solution 1:10) during 48 h with a daily solution changing. To reach neutral pH, the FSPs were washed with cold distilled water, then soaked for 24 h in butanol solution (10%, ratio 1:10) to eliminate fat and then thoroughly washed with cold water.

### 3.3. Collagen Extraction

Acid solubilised collagen and pepsin solubilised collagen were isolated from hound-smooth skin following the method proposed by Nagai and Suzuki [23] to which we introduced some modification as detailed in the following paragraph. All preparations were conducted in a cold room at 4 °C.

#### 3.3.1. Acid Extraction

To extract collagen, the pre-treated FSPs were suspended in a solution of acetic acid (0.5 M, ratio 1:10) for 3 days with a continuous stirring, filtered and the residue was subjected to a second extraction under the same conditions. Following this step NaCl was added to both supernatants to a final concentration of 2.0 M to precipitate collagen. The pellets were recovered by centrifugation (7000× *g*, during 1 h) then re-dissolved in acetic acid (0.5 M).

For purification, the resulting acetic acid solution was dialysed (bag cut-off of 14 kDa) against an acetic acid (0.1 M) solution, then against distilled water during 48 h in each case. The purified extract was thereafter lyophilized (Christ, Alpha 2–4 LD plus, Osterode am Harz, Germany), and the resulting collagen was referred as acetic ASC.

#### 3.3.2. Enzymatic Extraction

**Figure 9:**
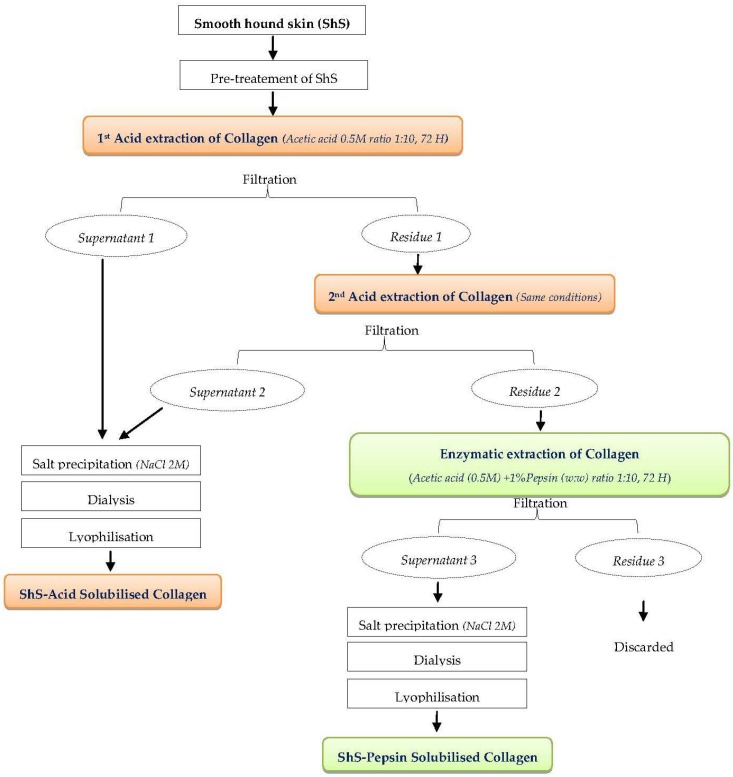
Collagen extraction process from smooth hound skin (ShS).

Un-dissolved materials (residue 2) resulting from the previous steps (Figure 9) were washed with cold distilled water and re-suspended in acetic acid (0.5 M) containing 1% pepsin (*w*/*w*) at a ratio of 1:10 (*w*/*v*) then incubated for 72 h at 4 °C. The filtrate was recovered in two steps for precipitation, dialysis and freeze drying as explained in ASC purification. The resulting collagen was called PSC. The collagens were stored at −20 °C until analyses.

### 3.4. Extracted Collagen Characterisation

#### 3.4.1. Sodium Dodecyl Sulfate Gel Electrophoresis

The determination of the collagen’s electrophoretic profiles was performed according to Laemmli [82] method. The extracted collagens ASC and PSC (1 mg/mL) were dissolved in 0.02 M sodium phosphate buffer (pH 7.2) containing urea (3.5 M) and sodium dodecyl sulfate (SDS = 1% *w*/*v*). The solubilised samples were then mixed with a Tris HCl buffer (0.5 M, pH 6.8) containing 10% (*w*/*v*) SDS, 50% (*v*/*v*) glycerol and 5% (*v*/*v*) b-mercaptoethanol (b-ME), at 1:1 (*v*/*v*) ratio. Electrophoresis was carried on a polyacrylamide gel made of 7.5% running gel and 4% stacking gel. Following 150 min of electrophoretic migration, the protein bands were stained with Coomassie brilliant blue R-250 (0.1%) in methanol and acetic acid (45%, 10% *v*/*v* respectively). After that the gel was finally distained with methanol and acetic acid (10%, 10% *v*/*v* respectively). High molecular weight markers (Biorad, CA, USA) were used to estimate collagen molecular weights. 

#### 3.4.2. Peptide Mapping

The ASC and PSC peptide mappings were determined according to the method of Kittiphattanabawon et al. [83] with a slight modification. To solubilise collagen, samples (6 mg) were suspended in 0.1 M sodium phosphate buffer (pH 7.2) containing 0.5% SDS, then heated at 100 °C for 5 min. The digestion of collagen in solutions (300 µL) was realized by adding 200 µL of Lysyl endopeptidase (from *Achromobacter lyticus*, 10 µg/mL buffer) and incubating at 37 °C for 5 min and 25 min. The proteolysis was stopped by incubating samples in boiling water for 3 min, then SDS-PAGE was realized using 12% running gel and 4% stacking gel.

#### 3.4.3. Viscosity Measurement

The ASC and PSC viscosities at different temperatures were measured according to Kimura and Ohno [84] procedure with some modification. The Ubbelohde viscometer (AVS 470, SI Analytics, Weilheim, Germany) was first filled with one of the collagen solution (1 mg/mL 0.1 M acetic acid), immersed in the water bath at 15 °C for 20 min; then temperature was increased stepwise up to 50 °C. The collagen solution viscosities were measured in each step of a 5 °C-temperature interval maintained for 20 min. The fractional viscosity of ASC and PSC at a designated temperature was calculated according to the formula below:

$$\mathrm{Fractional}\;\mathrm{viscosity}=\frac{\left(\mathrm{maximum}\;\mathrm{viscosity}-\mathrm{measured}\;\mathrm{viscosity}\right)}{\left(\mathrm{maximum}\;\mathrm{viscosity}-\mathrm{minimum}\;\mathrm{viscosity}\right)}$$

#### 3.4.4. Ultraviolet Spectrophotometric Analysis

The UV absorption spectrum of collagen samples was measured with an UV-spectrophotometer (LLG Labware, model unispec, Meckenheim, Germany). The spectrum of ASC and PSC samples (5 mg/mL) in acetic acid (0.5 M) were identified by scanning the wavelength between 190 and 500 nm. The baseline was set with 0.5 M acetic acid.

### 3.5. Preparation of Polymer Composite Films and Characterisation

To prepare the film forming solution, lyophilized PSC collagen was suspended at 0.1 (*w*/*v*) in 4% aqueous commercial vinegar solution with continuous stirring overnight at 4 °C.

The film-forming solution of chitosan extracted from shrimp waste (mean degree of deacetylation = 80%), was solubilised in a 4% aqueous commercial vinegar solution (1%, *w*:*v*) with an overnight continuous homogenisation at room temperature.

Once both polymer solutions were prepared, different films were prepared: pure CO, CH and mixture of collagen-chitosan solution with different ratios C_50_ (50% collagen, 50% chitosan), and C_75_ (25% collagen, 75% chitosan). For a homogeneous suspension, the collagen-chitosan solution was stirred continuously for at least 30 min at room temperature; the solution was then poured into Petri dishes and dried at room temperature. Finally, the dried films were peeled off manually and stored at maintained at 55% relative humidity in a desiccator before analysis.

#### 3.5.1. Thickness

The thickness of films was measured using a Thickness Tester (Thwing-Albert Instrument, ProGage, NJ, USA. Different locations on each film sample were used for determination of thickness. 

#### 3.5.2. Mechanical Properties

The film’s stress-strain properties (tensile strength TS and elongation at break point EAB) were measured in accordance to ISO 527-1 [85]. The test was performed using the universal testing machine (Lloyd Instruments Ltd, LRX plus Series, West Sussex, UK).

Films were cut to width 15 mm and conditioned at 23 ± 2 °C and ~50 ± 10% RH 25 ± 0.5 °C for 24 h before measurement. Initial grip separation distance was set to 30 mm and mechanical crosshead speed to 100 mm/min. 

#### 3.5.3. Water Solubility

The film water solubility was tested using the procedure of Gómez Estaca, et al. [86] with slight modification. Film sections of 1 × 1 cm^2^ (*n* = 6, in each case) were placed in an oven at 105 °C until constant weights were reached (W_i_). The films were then immersed in water for 24 h at room temperature with gentle shaken. After filtration, the residual films were dried again at 105 °C for 24 h (W_f_). The solubility of the films was calculated as:

$$\mathrm{Solubility}\;\left(\%\right)=\frac{\mathrm{Wi}\;-\mathrm{Wf}}{\mathrm{Wi}}\times 100$$

where, W_i_ = Initial weight of the film, W_f_ = Weight of the un-dissolved dried films residue.

#### 3.5.4. Light Barrier Properties

Using an UV-Visible Spectrophotometer (LLG Labware, model unispec, Meckenheim, Germany), the light barrier properties of the different polymer films were measured by exposing them to light absorption at wavelengths ranging from 200 nm to 800 nm. 

#### 3.5.5. Colour Properties

The colour of the films was determined using a CIE colorimeter (Konica Minolta Sensing, CR 410, Japan), and was expressed as: L*: luminance/brightness, a*: red/green and b*: yellow/blue. DE* (total difference in colour) was calculated using the equation below [87]:

$$\Delta E{*}^{\;}=\sqrt{{\left(\Delta L*\right)}^{2}+{\left(\Delta a*\right)}^{2}+{\left(\Delta b*\right)}^{2}}$$

where ∆L*, ∆a* and ∆b* are the differences between the corresponding colour parameter of the sample and the white standard.

#### 3.5.6. Fourier Transform Infrared Spectroscopy 

The FTIR analyses of collagens ASC and PSC extracted from smoothhound skin and of different films were performed using a Cary 630 FTIR spectrophotometer (Agilent Technologies, Santa Clara, CA, USA) within the wavenumber ranging between 4000 and 400 cm^−1^. In each case, the sample was placed directly on the FTIR spectrometer fitted with an Agilent diamond ATR sample.

#### 3.5.7. 1,1-Diphenyl-2-picrylhydrazyl Radical Scavenging Ability

The free radical-scavenging ability of films was measured as reported by Shimada, et al. [88] with slight modification. Films were cut in small pieces and dissolved in acetic acid solution (0.5 M) at 5 mg/mL, then 500 µL 0.1 mM DPPH solution was added to 500 µL of each film sample and kept in the dark for 30 min. Absorbance was measured at 571 nm and DPPH radical scavenging ability was calculated with the following equation:

$$\mathrm{DPPH}\left(\%\right)=\frac{\left(A\;\mathrm{control}\;–A\;\mathrm{sample}\right)}{A\;\mathrm{control}}\times 100$$

where A_sample_ and A_control_ were the absorbencies of sample and control group, respectively.

### 3.6. Statistical Analysis

The experiments (carried out at least in triplicate) were presented as mean ± standard deviation. Statistical interpretation of the results was performed by One way ANOVA and LSD (least significant differences) tests (using *p* < 0.05 level of significance to compare mean values) using the software SPSS 22.0 (SPSS 22.0 for Windows, SPSS Inc., Chicago, IL, USA).

## 4. Conclusions

In a first step the type of collagen extracted from the hound-smooth skin was identified. Thus, electrophoretic patterns revealed that both extracted ASC and PSC are mostly composed of α (α1, α2) and β-chains with low content of γ-chain suggesting that hound-smooth skin collagen should most likely be classified as type I collagen.

Fourier transform infrared investigations showed that the secondary structure and the triple helical structure of ASC and PSC were both maintained intact even after the enzymatic hydrolysis with pepsin. Similar denaturation temperatures were found for ASC and PSC (26.68 °C and 26.66 °C respectively). 

In a second step, collagen was used to elaborate film but with the incorporation of chitosan. The addition of chitosan increased the films mechanical strength and reduced its water solubility. The FTIR spectra clearly indicated that interactions between both polymers occurred and the secondary structure of collagen triple helix have been changed by the addition of chitosan. 

The addition of 50% chitosan into collagen films was sufficient to obtain an edible film with good mechanical properties, suitable solubility and antioxidant activity. Owing to its anti-UV properties, such collagen-chitosan film could be used as a protective material to preserve nutraceutical products. 

## Figures and Tables

**Table 1:** Thickness and mechanical properties (TS: Tensile strength and EAB: elongation at break) of CH and composite films C_50_ and C_75_.

	TS (MPa)	EAB (%)	Thickness (µm)
CH	70.52 ± 3.39 ^a^	4.25 ± 0.63 ^a^	17.15 ± 1.41 ^a^
C_50_	55.42 ± 8.6 ^b^	5.67 ± 0.51 ^b^	15.66 ± 1.63 ^b^
C_75_	66.28 ± 2.7 ^a,b^	4.49 ± 0.23 ^b^	16.07 ± 1.10 ^b^

All values are mean ± standard deviation; ^a,b^ different superscripts in the same column indicate significant differences (*p* < 0.05). CH: pure chitosan film; C_50_: Collagen-chitosan film 50%:50%, C_75_: Collagen-chitosan film 25%:75%.

**Table 2:** Table summarising tensile strength (TS) and elongation at break (EAB) values of biofilms reported in other works and commercial films.

Film	TS(MPa)	EAB (%)	Reference
Pure Chitosane	**70.52**	**4.25**	**Present study**
	51.04	2.25	[65]
	5.8	17.3	[54]
Pure Collagen	25.3	14.7	[54]
	2.3	2.2	[66]
Collagen: Chitosan C50	**55.42**	**5.67**	**Present study**
Collagen: Chitosan C75	**66.28**	**4.49**	**Present study**
Collagen from shark catfish skin: Chitosane	8.16	14.3	[67]
collagen from jumbo squid: chitosan (15:85)	35.5	12.3	[58]
Polyester	178	85	[68]
Polyvinyl chloride (PVC)	93	30	
Low-density polyethylene (LDPE)	13	500	
High-density polyethylene (HDPE)	26	300	
Hydroxypropyl cellulose	15	33	

**Table 3:** Color properties and solubility of collagen films where L*: luminance/brightness, a*: red/green, b*: yellow/blue and ΔE* : total difference in colour.

	L*	a*	b*	ΔE*	Film Solubility (%)
CO	97.93 ± 0.001 ^a^	0.07 ± 0.2 ^a^	2.13 ± 0.01 ^a^	0.35 ± 0.02 ^a^	32.14 ± 2.3 ^a^
C_50_	97.76 ± 0.002 ^b^	−0.09 ± 0.06 ^b^	2.63 ± 0.002 ^b^	0.85 ± 0.009 ^b^	24.55 ± 1.88 ^b^
C_75_	97.30 ± 0.0002 ^c^	−0.19 ± 0.09 ^c^	2.62 ± 0.003 ^b^	0.99 ± 0.008 ^c^	17.64 ± 2 ^c^
CH	97.26 ± 0.001 ^c^	−0.13 ± 0.03 ^d^	3.25 ± 0.03 ^c^	1.56 ± 0.08 ^d^	13.29 ± 1.02 ^d^

All values are mean ± standard deviation; ^a–d^ different superscripts in the same column indicate significant differences (*p* < 0.05). CO: pure collagen film; CH: pure chitosan film; C_50_: Collagen-chitosan film 50%:50%, C_75_: Collagen-chitosan film 25%:75%.

**Table 4:** Radical scavenging activity DPPH (1,1-diphenyl-2-picrylhydrazyl)of the different biofilms.

Films	DPPH (%)	Reference
Pure Collagen CO	**30.88 ±0.03 ^a^**	**This study**
Cuttlefish Gelatin	30.99	[78]
Chitosan CH	**24.13± 0.75 ^b^**	**This study**
	0.17	[79]
Collagen: Chitosan C50	**23.91± 1.15 ^b^**	**This study**
Collagen: Chitosan C75	**19.77± 0.25 ^b^**	
Gelatin film + henna extract	61.86	[78]
Chitosan + Eucalyptus globulus essential oil	23.03–43.62	[80]
Chitosan + xanthan gum + fish protein hydrolysate	1.7–2.46	[79]

All values are mean ± standard deviation; ^a,b^ different superscripts in the same column indicate significant differences (p < 0.05). C50: Collagen-chitosan film 50%:50%, C75: Collagen-chitosan film 25%:75%.
